# Mental well-being of the general population: direct and indirect effects of socioeconomic, relational and health factors

**DOI:** 10.1007/s11136-021-02813-5

**Published:** 2021-04-13

**Authors:** Natalia Soldevila-Domenech, Carlos G. Forero, Itxaso Alayo, Jordina Capella, Joan Colom, Davide Malmusi, Anna Mompart, Philippe Mortier, Beatriz Puértolas, Néstor Sánchez, Anna Schiaffino, Gemma Vilagut, Jordi Alonso

**Affiliations:** 1 Health Services Research Group, Epidemiology and Public Health Program, Hospital del Mar Medical Research Institute (IMIM), Carrer del Doctor Aiguader, 88, PRBB Building, 08003 Barcelona, Spain; 2 Integrative Pharmacology and Systems Neurosciences Research Group, Neurosciences Research Program, Hospital del Mar Medical Research Institute (IMIM), Carrer del Doctor Aiguader, 88, PRBB Building, 08003 Barcelona, Spain; 3 Department of Experimental and Health Sciences, Pompeu Fabra University (UPF), Carrer del Doctor Aiguader, 88, PRBB Building, 08003 Barcelona, Spain; 4 School of Medicine, Universitat Internacional de Catalunya (UIC), Barcelona, Spain; 5 CIBER en Epidemiología y Salud Pública (CIBERESP), Av. Monforte de Lemos, 3-5, 28029 Madrid, Spain; 6 Programme on substance Abuse, Agency of Public Health of Catalonia, Carrer Roc Boronat, 81-95, 08005 Barcelona, Spain; 7 Ajuntament de Barcelona, Barcelona, Spain; 8 Direcció General de Planificació en Salut, Departament de Salut, Generalitat de Catalunya, Travessera de les Corts, 131-159, 08028 Barcelona, Spain; 9 Institut Català D’Oncologia, Gran Via de l’Hospitalet 199-203, 08908 l’Hospitalet de Llobregat, Spain; 10 Direcció General d’Ordenació i Regulació Sanitàries, Departament de Salut, Generalitat de Catalunya, Travessera de les Corts, 131-159, 08028 Barcelona, Spain

**Keywords:** Well-being, WEMWBS, Mental health, Health determinants, Health survey, Directed acyclic graphs

## Abstract

**Purpose:**

The aim of this study was to analyse the association between individual mental well-being and social, economic, lifestyle and health factors.

**Methods:**

Cross-sectional study on a representative sample of 13,632 participants (> 15y/o) from the Catalan Health Interview Survey 2013–2016 editions. Mental well-being was assessed with the Warwick–Edinburg Mental Well-being Scale (WEMWBS). Linear regressions were fitted to associate well-being and sociodemographic, relational, lifestyle and health variables according to minimally sufficient adjustment sets identified using directed acyclic graphs. Predictors entered the model in blocks of variable types and analysed individually. Direct and total effects were estimated.

**Results:**

Health factors significantly contributed to mental well-being variance. Presence of a mental disorder and self-reported health had the largest effect size (eta^2^ = 13.4% and 16.3%). The higher individual impact from a variable came from social support (*β* = − 12.8, SE = 0.48, eta^2^ = 6.3%). A noticeable effect gradient (eta^2^ = 4.2%) from low to high mental well-being emerged according to economic difficulties (from *β* = 1.59, SE = 0.33 for moderate difficulties to *β* = 6.02 SE = 0.55 for no difficulties). Younger age (*β* = 5.21, SE = 0.26, eta^2^ = 3.4%) and being men (*β* = 1.32, SE = 0.15, eta^2^ = 0.6%) were associated with better mental well-being. Direct gender effects were negligible.

**Conclusions:**

This study highlights health and social support as the most associated factors with individual mental well-being over socioeconomic factors. Interventions and policies aimed to these factors for health promotion would improve population mental well-being.

**Supplementary Information:**

The online version contains supplementary material available at 10.1007/s11136-021-02813-5.

## Plain English summary

Mental well-being is typically understood as ‘feeling good’ and ‘functioning well’ and it is considered an indicator of societal progress. However, there is a lack of knowledge about its risk and protective factors. The study of drivers of mental well-being is important to understand how mental health operates in the population beyond mental illness. In this study, we explored the impact of demographic, socioeconomic, relational, lifestyle and health factors on the mental well-being, in a representative general population sample of 13,632 individuals. This study indicates that mental well-being is sensitive to demographic and socioeconomic factors such as gender, age, education, employment and economic difficulties, as well as, to relational and health factors. Adverse health factors and self-reported health are most strongly associated with mental well-being over socioeconomic factors, which would have an indirect impact on mental well-being. Finally, the lack of social support appears as a critical risk factor of decreased mental well-being. Findings from this study suggest that interventions and policies aimed for health promotion would improve population mental well-being.

## Introduction

The evaluation of well-being at the individual level receives increasing attention for its potential impact on health, economy and societal progress [1, 2]. The World Health Organisation (WHO) emphasizes that ‘there is no health without mental health’, which includes aspects of psychological, emotional and social well-being [3]. Mental well-being, defined as ‘feeling good’ and ‘functioning well’ [4], may generate resilience to mental and physical illnesses, boost educational achievement, enhance performance in the workplace and increase longevity in the general population [5, 6]. Consequently, promoting mental well-being may also be a useful approach to health promotion and disease prevention [7]. To achieve this, we need more evidence on its risk and protective factors [8, 9].

In the WHO model of social determinants of health and well-being [10], the socioeconomic and political context gives rise to structural determinants of health (gender, age, ethnicity, social class), responsible of health inequalities by influencing how people live and work and affecting the exposure to risk and protective factors over the life course. The socioeconomic status (education, employment and money) puts people in economic difficulties at “risk of risk” [11], conditioning proximal determinants of mental well-being, including relational factors (e.g. social support), health factors (e.g. physical and mental disorders, disability) and perceived health [12]. Similarly, the pathway from lifestyle factors to well-being would act as risk or protective factors of mental and physical disorders [13]. Among relational factors, functional social support provides emotional, instrumental and informational resources, which have been identified as affected by unemployment, retirement or economic difficulties [11, 12]. Social support may directly impact mental well-being by promoting the sense of belonging, enhancing self-realization and increasing coping abilities [14, 15].

Some authors suggest that mental disorders may have stronger effects on mental well-being than physical disorders due to the higher personal uncertainty and compromised ability to display adaptive conducts associated with such conditions [16]. Also because of adaptation, recent acute health problems may have a higher impact on well-being than long-term chronic conditions [17, 18]. Disability would mediate the impact of physical and mental conditions on perceived health [19], and could also impact mental well-being directly due to its pervasive effects on major areas of everyday life. Self-perceived health status reflects both the actual physical condition and its emotional impact on general living conditions [20]. Actually, there is evidence that the relative effect of self-reported health on well-being is larger than that of income and social relationships [15].

Identifying the causal direction among these factors is not straightforward. Also, to date, determinants of mental well-being as an outcome have been researched with various instruments more focussed on mental disorders rather than on positive aspects of mental health. In recent years, the Warwick Edinburgh Mental Well-being Scale (WEMWBS) has emerged as an increasingly popular individual-level measure of positive mental well-being [21]. Studies using WEMWBS have shown that mental well-being does not mirror the traditional gradients reported for mental illness [22] and that its predictors differ from those of psychopathology [23], making it a potentially informative instrument for analysing population mental well-being.

A better comprehension of the drivers of positive mental health would increase our understanding of how mental health operates in the population beyond mental illness. Such understanding would boost our ability to monitor public health policies and interventions for promoting population health. In this study, we aim to identify potential determinants of population mental well-being, as measured with the WEMWBS, using data from a large representative population sample of the Catalan population (Spain). Specifically, and based on the general WHO model of health determinants, we intend to estimate the associations between mental well-being and demographic, socioeconomic, relational, lifestyle and health-related blocks of variables, as well as analysing the direct and indirect effect of each specific variable on mental well-being. We hypothesized (1) the existence of social inequalities in the distribution of mental well-being; (2) a higher contribution of relational and health factors to the mental well-being over lifestyle, socioeconomic and demographic factors; and (3) a substantial positive contribution of health factors and self-perceived health on the indirect effect of each variable on mental well-being.

## Methods

### Design, information sources and study population

Repeated cross-sectional design using data from 7 biannual waves of the Catalan Health Interview Survey (ESCA) during 2013–2016 (*N* = 13,632). Each wave ensues an independent representative sample of the general population (over 15 years old) of Catalonia, a north-eastern region of Spain (7.5 million inhabitants). The sampling frame is the non-institutionalized adult population in the Population Registry of Catalonia from the Catalan Institute of Statistics (IDESCAT). Individuals are selected through stratified three-stage random sampling with different probabilities within strata. The ESCA has the rank of official statistics by the Government of the Catalonia, so participants must answer the survey in a complete and truthful way (Law 23/1998, December 30, of statistics of Catalonia). Data are obtained through face-to-face interviews by trained interviewers at the respondent’s home using computer-assisted personal interviewing (CAPI). Interview time takes about 45 min [24].

Study’s population features were as follows: 50.9% women; mean age 47.4 years; 21.3% had up to primary studies; 14.4% were born in non-high income countries; 10.7% were unemployed; 15.1% were retired, and 20.6% had difficulties in making monthly ends meet. Low social support was present in 2.5% women and 1.7% men. Regarding lifestyle factors, 14.7% were obese, and 25.5% were current smokers. As for health factors, 17.0% reported life-time history of at least one mental disorder (22.9% women and 10.9% men); 63.9% reported at least one physical disorder (70.6% women and 56.9% men); 8.0% reported lack of autonomy, and 3.5% reported “poor” perceived health status. See Table 2 for detailed sample characteristics.

### Variables

#### Mental well-being

The primary outcome was mental well-being as measured with Spanish and Catalan versions of the WEMWBS [25, 26]. It is a unidimensional measure of mental well-being in the previous two weeks [21] using 14 Likert-scaled positively-worded items (e.g. “I’ve been feeling optimistic about the future”), with five categories from “None of the time” to “All of the time”. Sumscore ranges 14–70, with higher scores indicating higher levels of mental well-being. The Spanish and English versions of the WEMWBS have shown high internal consistency and reliability (Cronbach’s alpha higher than 0.93 and 0.91, respectively) and adequate discriminative capacity between socioeconomic groups and health-related conditions to perform studies about health, social or economic inequalities [21, 25].

#### Predictive factors of mental well-being

We explored the WEMWBS relationship with six blocks of factors:

1. *Demographic factors*: Sex; Age; and Country of origin, categorized as Spain, high income and other according to the Organisation for Economic Co-operation and Development (OECD) classification [27].
2. *Socioeconomic factors:* Educational level (primary or less, secondary, and higher); Employment status (student; employed; unemployed; housework; retired; other); Economic difficulties, obtained from a question about the presence of family economic difficulties to make monthly ends meet; and Social class of the household reference person, based on occupation [28]: class I (directors, managers and university professionals), class II (intermediate occupations and self-employed workers), class III (manual workers) and not classifiable (never worked and living alone).
3. *Relational factors*: Perceived social support was assessed with the DUKE-UNC-11 Functional Social Support Scale, covering confidant support (e.g. chances to talk about work or money problems), and affective support (e.g. displays of affection, love, and empathy) [29]. It has 11 Likert-scaled items, with total score ranging 11–55 points, with higher scores indicating lower social support. In the Spanish validation of the DUKE-UNC-11, a cut-off point at the 15th percentile was chosen to categorize subjects as ‘low’ (≥ 32 points) and ‘adequate’ (< 32 points) social support [30]. The Spanish version of the DUKE-UNC-11 shows high internal consistency and reliability (Cronbach’s alpha of 0.90) [30].
4. *Lifestyle factors*: Body mass index (BMI, kg/m^2^) categorized as: underweight (BMI < 18.5), normal (18.5 ≥ BMI < 25), overweight (25 ≥ BMI < 30), and obesity (BMI ≥ 30); hours of sleep; and smoking status.
5. *Health factors:* Life-time history of mental disorders (anxiety, depression or other mental disorders); lifetime history of long-term (≥ 6 months) physical disorders from a list of 28 [24]; and lack of autonomy, as need of help to perform routine activities due to a health problem.
6. *Self-Perceived health*: Using the question: “In general how would you say your health is?” in a 5-point ordinal scale (from Excellent to *Poor*).

### Statistical analysis

We computed descriptive statistics and standard errors (SE) of WEMWBS scores and study variables, stratified by sex. We tested score differences between categories using one-way analysis of variance (ANOVA), adjusting for multiple comparisons using Benjamini–Hochberg procedure [31] at false discovery rate of 5%. Effect sizes were computed as either Cohen’s d with cut-offs of 0.2 (small), 0.5 (medium), 0.8 (large) and 1.2 (very large) [32, 33] or, in regression models, as eta-squared with cut-offs of 1% (small), 6% (moderate) and 14% (large) [34].

To explore the contribution of each block on mental well-being, we fitted multivariable linear block regression models. Models were devised in a block-nested manner, added from distal to proximal. The first block included demographic factors; the following models added blocks consecutively: socioeconomic factors; relational factors; lifestyle factors; health factors and perceived health. Models increase in explained variance was assessed by block using R-squared, and tested using F-test at 0.05 nominal level. The final full model included six blocks and all variables.

We estimated multivariable adjusted linear regression models for each predictor variable. To avoid overadjustment bias [35], we used directed acyclic graph (DAG) methodology to determine the variables for which it is sufficient to control to obtain unbiased estimates of causal effects [36]. DAGs are especially useful for using a priori knowledge (i.e. they use no actual data) on the relation between variables, thus displaying graphically explicit and transparent assumptions based on former available evidence. Relationships are “directed” as variables imply a causal sequence, which are represented by arrows. When there is insufficient evidence to exclude a potential effect between variables, such relationship is also assumed and so is reflected in the DAG. Once a relationship framework is developed, DAGs apply algebraic methods (Pearl’s back- and single-door criterion [37–39]) to trim model-biasing pathways, yielding two minimally sufficient adjustment sets (MSAS) for each predictor, one for estimating the total effect and the other for the direct effects (unmediated paths directly connecting a predictor variable and an outcome). The MSAS represent the lowest number of covariates needed for adjusting after excluding irrelevant variables that can actually cause bias when included in the multivariable regression model [40]. Once total and direct effects are estimated, it is possible to decompose total effects in direct and indirect (i.e. remaining effects through paths including mediators and other variables).

**Fig. 1:**
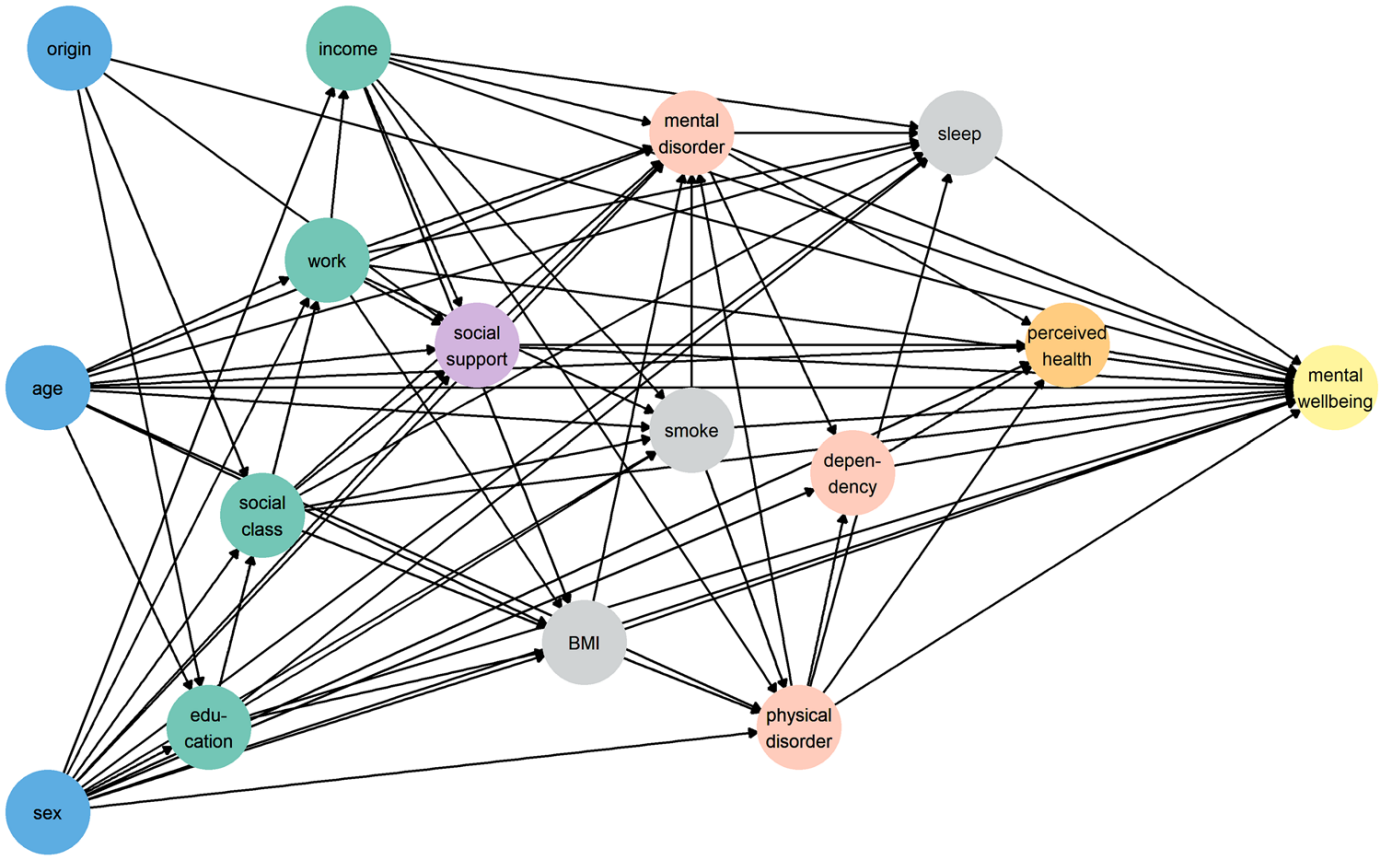
Directed acyclic graph (DAG-1). Variable names are abbreviated: *origin* country of origin; *education* educational level; *work* employment status; *income* family economic difficulties; *BMI* body mass index; *smoke* smoking status; *sleep* hours of sleep; *dependency* lack of autonomy. Node colours represent the group to which each variable belongs: demographic factors (in blue), socioeconomic factors (in green), relational factors (in purple), lifestyle factors (in grey), health factors (in red), and self-reported health (in orange). (Color figure online)

We devised a first directed acyclic graph (DAG-1) (Fig. 1), where we assumed that all predictor variables were directly related to mental well-being. Table 1 details on other assumed relationships between covariates. The two suitable MSAS identified from the DAG-1 were entered in two different linear regression models for each predictor, to estimate total and direct effects. Finally, to study whether health factors and self-reported health mediated the effect of all other factors on mental well-being, a second DAG (DAG-2) was devised (Supplementary Figure 2), identical to DAG-1 but excluding health factors and self-reported health from the pathways to well-being. For each predictor, the rate between direct effect differences between DAG-1 and DAG-2 over indirect effects from DAG-1 was computed to study the contribution of health factors and self-perceived health on the indirect effect of each variable on mental well-being. All analyses were adjusted by survey year.

**Table 1:** Assumed relationships between covariables of mental well-being when developing the DAG

Directed edges		Potential bidirectionality*	References
From	To		
Sex	All variables except age and country of origin	No	[61, 66, 67]
Age	All variables except sex and country of origin	No	
Country of origin	Education	No	
	Social class	No	
	Social support	No	
Education	Employment status	Yes ( +)	[68, 69]
	Social class	Yes (+ +)	
	BMI	No	
	Smoking status	No	
	Hours of sleep	No	
	Mental disorder	Yes ( +)	
Social class	Employment status	Yes (+ +)	[70–72]
	Social support	No	
	BMI	No	
	Smoking status	No	
	Hours of sleep	No	
	Physical disorder	No	
	Mental disorder	No	
Employment status	Economic difficulties	Yes (+ +)	
	Social support	Yes ( +)	
	BMI	Yes ( +)	
	Smoking status	Yes ( +)	
	Hours of sleep	Yes ( +)	
	Mental disorder	Yes ( +)	
Economic difficulties	Social support	No	
	BMI	No	
	Smoking status	No	
	Hours of sleep	No	
	Physical disorder	Yes ( +)	
	Mental disorder	Yes ( +)	
Social support	Mental disorder	Yes (+ +)	[14, 55, 73]
	Perceived health	Yes ( +)	
Body mass index (BMI)	Physical disorder	Yes (+ + +)	[61, 66]
	Mental disorder	Yes (+ + +)	
Smoking status	Physical disorder	Yes (+ +)	
	Mental disorder	Yes ( +)	
Physical disorder	Hours of sleep	Yes ( +)	[19, 60, 74, 75]
	Mental disorder	Yes (+ + +)	
	Lack of autonomy	Yes ( +)	
	Perceived health	Yes (+ +)	
Mental disorder	Hours of sleep	Yes (+ +)	
	Lack of autonomy	Yes ( +)	
	Perceived health	Yes (+ +)	
Lack of autonomy	Perceived health	Yes (+ +)	

*Threatens to the face-validity (plausibility of the posited relationship) are assessed according to evidence, and “Yes” is given in the cases where reverse directionality is plausible. In such cases, the chance of reverse causality is qualitatively evaluated with (+ , +  + , +  + +), indicating less or more probability of bidirectionality, respectively

Inverse probability sampling weights and post-stratification weights were applied to achieve representativeness in terms of geographic areas, age, sex, and strata sizes. Weights were normalized to the total sample size so that the samples of each year had equal weights in the analyses. Missing values were < 1.6% so each specific analysis was performed on individuals with complete information on the variables involved. DAGs and MSAS for total and direct effects were devised using the ‘dagitty’ package in R [41]. Data management were done with Stata version 13 [42], and statistical analyses with R version 3.5.2 [43].

### Ethics

The ESCA has the rank of official statistics carried out by the Government of the Catalonia region, and it must ensure the confidentiality of the data (Law 23/1998, December 30th of statistics of Catalonia).

## Results

Table 2 shows descriptive statistics of mental well-being across categories of studied variables (see Supplementary Figure 1 for a visual representation of effect sizes stratified by sex). As expected from the large sample size, all variables showed significant differences in WEMWBS scores. Demographic, socioeconomic and lifestyle factors had small effects on mental well-being. Women presented slightly lower mental well-being than men (58.2 vs 59.5, Cohen’s *d* = 0.13). Negative effect gradients emerged according to age (from 60.2 in the 15–44y/o group to 55.0 over 75y/o) and family economic difficulties from least to most difficulties (ranging from 61.7 to 54.9). Those who declared low social support had lower WEMWBS scores (44.0 vs 59.2, Cohen’s *d* = − 1.54). Perceived health status also yielded a negative gradient of WEMWBS scores, from 63.4 for excellent health to 46.1 for poor health. Small differences in effect sizes appeared when stratifying by sex: being student, employed or having a mental disorder had larger effect in men than in women. On the contrary, lack of autonomy had higher effect in women than in men (Cohen’s *d* = 0.76 and 0.64, respectively).

**Table 2:** Distribution of sample characteristics (*N*, %) and description of WEMWBS scores (mean, SE) across categories of study variables

Variable	Category	Total (*N*^#^ = 13,632)		Women (*N*^#^ = 6814)		Men (*N*^#^ = 6818)	
		*N*^#^ (%)	WEMWBS score Mean* (SE)	*N*^#^ (%)	WEMWBS score Mean* (SE)	*N*^#^ (%)	WEMWBS score Mean* (SE)
Sex	Men	6818 (49.1)	59.5 (0.12)				
	Women	6814 (50.9)	58.2 (0.13)				
Age (years)	15—44	6372 (48.5)	60.2 (0.11)	3113 (46.5)	59.9 (0.16)	3259 (50.7)	60.5 (0.15)
	45—64	4311 (31.2)	58.3 (0.16)	2141 (30.9)	57.6 (0.23)	2170 (31.6)	59.1 (0.21)
	65—74	1377 (10.5)	57.6 (0.30)	720 (11.1)	57.0 (0.43)	657 (10.0)	58.4 (0.41)
	≥ 75	1572 (9.7)	55.0 (0.31)	840 (11.5)	54.4 (0.42)	732 (7.8)	56.0 (0.42)
Country of origin	Spain	11,406 (83.7)	58.7 (0.10)	5700 (84.0)	58.1 (0.14)	5706 (83.3)	59.4 (0.13)
	High income	287 (1.9)	60.0 (0.50)	143 (1.7)	59.8 (0.68)	144 (2.1)	60.2 (0.73)
	Other	1936 (14.4)	59.6 (0.22)	970 (14.3)	59.0 (0.32)	966 (14.6)	60.2 (0.30)
Educational level	Up to Primary	3085 (21.3)	56.2 (0.21)	1588 (22.8)	55.5 (0.30)	1497 (19.7)	57.0 (0.30)
	Secondary	7926 (57.8)	59.3 (0.11)	3808 (55.2)	58.7 (0.17)	4118 (60.6)	59.9 (0.15)
	Higher	2615 (20.8)	60.4 (0.16)	1415 (22.0)	60.0 (0.23)	1200 (19.7)	60.9 (0.21)
Social class	I	2623 (20.5)	60.1 (0.16)	1274 (20.2)	59.8 (0.23)	1349 (20.9)	60.5 (0.22)
	II	2256 (17.3)	59.2 (0.20)	1207 (18.2)	58.7 (0.30)	1049 (16.4)	59.7 (0.26)
	III	8304 (58.8)	58.5 (0.12)	4005 (56.9)	57.8 (0.17)	4299 (60.8)	59.2 (0.16)
	Has never worked	294 (2.0)	54.1 (0.77)	236 (3.1)	53.3 (0.80)	58 (0.9)	57.1 (1.95)
Employment status	Student	1150 (8.7)	61.0 (0.24)	571 (8.3)	60.4 (0.36)	579 (9.0)	61.5 (0.32)
	Employed	6857 (50.4)	60.4 (0.10)	3158 (46.0)	59.8 (0.16)	3699 (55.0)	60.9 (0.13)
	Unemployed	1330 (10.7)	57.1 (0.29)	551 (9.0)	57.0 (0.45)	779 (12.6)	57.1 (0.38)
	Housework	1401 (10.3)	56.9 (0.30)	1401 (20.1)	56.9 (0.30)	0 (0.0)	
	Retired	2263 (15.1)	57.5 (0.23)	842 (12.2)	56.7 (0.39)	1421 (18.1)	58.1 (0.28)
	Other conditions	592 (4.6)	51.7 (0.55)	254 (3.8)	50.0 (0.84)	338 (5.4)	53.0 (0.72)
Family economic difficulties to make monthly ends meet	Great difficulty	851 (7.3)	54.9 (0.44)	433 (7.4)	54.3 (0.62)	418 (7.1)	55.6 (0.62)
	Difficulty	1603 (13.3)	56.5 (0.27)	851 (14.0)	55.7 (0.38)	752 (12.6)	57.5 (0.38)
	Some difficulty	3216 (24.9)	58.4 (0.18)	1644 (25.4)	57.9 (0.25)	1572 (24.4)	58.9 (0.24)
	Some ease	5533 (37.7)	59.7 (0.12)	2687 (36.6)	59.1 (0.17)	2846 (38.8)	60.3 (0.15)
	Ease	2135 (14.8)	61.1 (0.19)	1071 (14.7)	60.4 (0.28)	1064 (14.8)	61.9 (0.25)
	Great ease	294 (2.1)	61.7 (0.54)	128 (1.9)	61.6 (0.88)	166 (2.4)	61.8 (0.66)
Social support	Low	263 (2.1)	44.0 (0.81)	157 (2.5)	43.3 (1.05)	106 (1.7)	45.1 (1.26)
	Adequate	13,369 (97.9)	59.2 (0.08)	6657 (97.5)	58.6 (0.12)	6712 (98.3)	59.8 (0.11)
BMI	Underweight	330 (2.4)	58.4 (0.60)	256 (3.7)	58.6 (0.68)	74 (1.1)	57.7 (1.29)
	Normal weight	6426 (47.6)	59.6 (0.12)	3537 (52.1)	59.1 (0.16)	2889 (43.0)	60.2 (0.17)
	Overweight	4642 (33.7)	58.8 (0.15)	1849 (26.9)	57.8 (0.24)	2793 (40.7)	59.5 (0.18)
	Obesity	2041 (14.7)	57.0 (0.25)	1035 (14.9)	56.2 (0.36)	1006 (14.6)	58.0 (0.34)
Hours of sleep	< 6 h	1064 (8.4)	55.6 (0.37)	619 (9.7)	55.0 (0.48)	445 (7.0)	56.4 (0.57)
	6-8 h	11,231 (82.6)	59.3 (0.09)	5549 (81.4)	58.7 (0.13)	5682 (83.8)	59.9 (0.12)
	> 8 h	1320 (8.9)	58.1 (0.33)	635 (8.7)	57.4 (0.50)	685 (9.1)	58.7 (0.44)
Smoking status	Current smoker	3488 (25.5)	58.6 (0.17)	1419 (20.7)	57.8 (0.29)	2069 (30.5)	59.1 (0.22)
	Ex-smoker	2551 (19.5)	59.3 (0.20)	897 (14.4)	59.5 (0.31)	1654 (24.8)	59.2 (0.25)
	Non-smoker	7593 (55.0)	58.9 (0.12)	4498 (65.0)	58.1 (0.16)	3095 (44.7)	60.0 (0.16)
Mental disorder	Yes	2170 (17.0)	51.8 (0.27)	1484 (22.9)	52.2 (0.32)	686 (10.9)	51.0 (0.48)
	No	11,462 (83.0)	60.3 (0.08)	5330 (77.1)	60.0 (0.12)	6132 (89.1)	60.6 (0.11)
Physical disorder	Yes	8798 (63.9)	57.6 (0.12)	4847 (70.6)	57.2 (0.16)	3951 (56.9)	58.2 (0.17)
	No	4834 (36.1)	61.0 (0.11)	1967 (29.4)	60.6 (0.17)	2867 (43.1)	61.3 (0.15)
Lack of autonomy	No	12,525 (92.0)	59.7 (0.08)	6160 (90.1)	59.3 (0.12)	6365 (94.0)	60.1 (0.11)
	Yes	1107 (8.0)	49.3 (0.39)	654 (9.9)	48.3 (0.50)	453 (6.0)	51.0 (0.62)
Perceived health status	Excellent	1044 (8.2)	63.4 (0.21)	439 (6.8)	63.2 (0.33)	605 (9.6)	63.5 (0.27)
	Very good	4410 (32.4)	60.7 (0.11)	2122 (30.8)	60.4 (0.17)	2288 (34.0)	60.9 (0.16)
	Good	5678 (40.9)	59.4 (0.12)	2848 (41.2)	59.1 (0.17)	2830 (40.6)	59.8 (0.17)
	Fair	2032 (15.0)	54.0 (0.26)	1127 (17.0)	53.4 (0.36)	905 (13.0)	54.8 (0.38)
	Poor	467 (3.5)	46.1 (0.61)	277 (4.2)	45.5 (0.78)	190 (2.8)	47.1 (0.95)

*BMI* body mass index, *N* number, *SE* standard error

*All variables have a significant effect on mental well-being (one-way ANOVA; *p* < 0.05) after the Benjamini–Hochberg correction with false discovery rate 0.05

^#^Unweighted *N*

Table 3 displays model fit and explained variance in block regression models. All blocks had impact on mental well-being. By block, socioeconomic factors explained 9.8% of mental well-being variance; relational factors explained 6.3%, and lifestyle factors explained only 2.2%. Health factor block had the most substantial contribution to mental well-being with 20.4% of explained variance, and perceived health status explained 16.3%. Sequential block entry showed that, after adjusting by year of survey and demographic factors, the incremental contribution of socioeconomic factors was 7.8%. Successive block contribution was: relational factors 4.4%; lifestyle factors 0.1%; health factors 10.0%, and self-reported health 2.9%. The full model with all variables explained 29.4% of mental well-being variance. Similar results were observed when stratifying by sex (results available upon request).

**Table 3:** Model fit and % explained variance by (a) individual blocks of factors and (b) incremental block effects when blocks entered sequentially

	Block effect	Incremental block effects	
	Explained variance (%)	Total explained variance (%)	Increase in explained variance (%)
Year of survey	0.4	0.4	–
Demographic factors	3.8	4.2	3.8
Socioeconomic factors	9.8	12.0	7.8
Relational factors	6.3	16.4	4.4
Lifestyle factors	2.2	16.5	0.1
Health factors	20.4	26.5	10.0
Perceived health*	16.3	29.4	2.9

Each model contained all variables within each block

All models are significant (*F* statistic *p* < 0.001). The % variance is computed as, adjusted *R*^2^*100 (%)

*The final model with the seven blocks factors contains all studied variables

Table 4 includes regression coefficients for total, direct and indirect effects of individual variables adjusted by MSAS from DAG-1. Supplementary Figure 2 summarizes the variables used to build each model. As hypothesized, the largest effect sizes were those of health and relational factors. Low social support had a moderate association with well-being (eta^2^ = 6.3%), implying 12 points less mental well-being. Self-reported health had the highest effect (eta^2^ = 16.3%) with a 10-point difference from excellent to poor health. Being dependent and having a mental disorder also implied lower well-being (eta^2^ = 10.6% and 13.4%, respectively). Except for employment (eta^2^ = 6.4%), demographic, socioeconomic and lifestyle factors showed low association with mental well-being.

**Table 4:** Total and direct effects of each variable on mental well-being in all population

				Total effects				Direct effects			
				*β*	SE	*P*	eta^2^ (%)	*β*	SE	*P*	eta^2^ (%)
Demographic factors	Sex (Ref: Women)		Men	1.32	0.15	< 0.001	0.6	− 0.05	0.14	0.741	0.6
	Age (years) (Ref: ≥ 75)		15–44	5.21	0.26	< 0.001	3.4	1.33	0.36	< 0.001	3.4
			45–64	3.35	0.27	< 0.001		1.24	0.33	< 0.001	
			65–74	2.67	0.32	< 0.001		1.47	0.29	< 0.001	
	Country of origin (Ref: Spain)		High income	1.36	0.54	0.012	0.2	0.68	0.50	0.173	0.2
			Other	0.90	0.21	< 0.001		1.01	0.21	< 0.001	
Socioeconomic factors	Educational level		Secondary	1.99	0.20	< 0.001	2.8	0.84	0.19	< 0.001	1.7
	(Ref: Primary)		Higher	3.05	0.24	< 0.001		0.71	0.26	0.006	
	Social class		I	0.68	0.22	0.002	1.2	− 0.42	0.20	0.037	1.2
	(Ref: III)		II	0.16	0.21	0.444		− 0.46	0.18	0.013	
			Never worked	− 3.75	0.52	< 0.001		− 2.58	0.50	< 0.001	
	Employment status (Ref: Unemployed)		Student	3.35	0.34	< 0.001	6.5	1.67	0.32	< 0.001	6.4
			Employed	3.16	0.24	< 0.001		1.81	0.23	< 0.001	
			Housework	2.34	0.36	< 0.001		1.50	0.33	< 0.001	
			Retired	2.65	0.39	< 0.001		1.90	0.37	< 0.001	
			Other	− 4.11	0.41	< 0.001		− 1.81	0.38	< 0.001	
	Family economic difficulties to make monthly ends meet (Ref: Great difficulty)		Difficulty	1.59	0.33	< 0.001	4.2	0.60	0.31	0.05	4.2
			Some difficulty	3.14	0.30	< 0.001		1.52	0.29	< 0.001	
			Some ease	4.36	0.30	< 0.001		2.24	0.29	< 0.001	
			Ease	5.64	0.33	< 0.001		3.70	0.32	< 0.001	
			Great ease	6.02	0.55	< 0.001		3.98	0.52	< 0.001	
Relational factors	Social support (Ref: Adequate)		Low	− 12.80	0.48	< 0.001	6.3	− 9.97	0.45	< 0.001	5.9
Lifestyle factors	BMI (Ref: Obesity)		Underweight	− 0.38	0.50	0.446	1.0	− 0.80	0.46	0.086	1.0
			Normal weight	0.78	0.22	< 0.001		0.34	0.20	0.097	
			Overweight	0.72	0.22	< 0.001		0.37	0.20	0.068	
	Hours of sleep (Ref: < 6 h)		6-8 h	1.37	0.24	< 0.001	1.5	1.37	0.24	< 0.001	1.5
			> 8 h	1.51	0.32	< 0.001		1.51	0.32	< 0.001	
	Smoking status (Ref: Smoker)		Ex-smoker	0.96	0.22	< 0.001	0.1	0.70	0.20	< 0.001	0.1
			Non-smoker	0.65	0.18	< 0.001		0.31	0.16	0.058	
Health factors	Mental disorder (Ref: Presence)		Absence	6.36	0.19	< 0.001	13.4	4.89	0.19	< 0.001	13.3
	Physical disorder (Ref: Presence)		Absence	2.15	0.16	< 0.001	3.6	0.40	0.15	0.006	3.5
	Lack of autonomy (Ref: Presence)		Absence	7.84	0.25	< 0.001	10.6	5.16	0.26	< 0.001	10.5
Self-reported health	Perceived health (Ref: Poor)		Excellent	10.07	0.45	< 0.001	16.3	10.07	0.45	< 0.001	16.3
			Very good	7.73	0.40	< 0.001		7.73	0.40	< 0.001	
			Good	7.14	0.38	< 0.001		7.14	0.38	< 0.001	
			Fair	4.17	0.38	< 0.001		4.17	0.38	< 0.001	

Regression coefficients represent WEMWBS change relative to the variable reference category. Each variable has been adjusted by its minimally sufficient adjustment set (MSAS) identified with the DAG-1 (see Fig. 1 and Supplementary Figure 2), and by year of survey

*β* regression coefficient, *SE* standard error, *eta2 (%)* eta squared*100, *BMI* body mass index, *Ref* reference category

Noticeably, there was no direct association between sex and mental well-being (*β* = − 0.05, SE 0.14). The direct effect of smoking habits was also negligible (eta^2^ = 0.1%). Age, education, social class, social support, BMI, hours of sleep, mental disorder, lack of autonomy and perceived health status had higher effects in women than in men; the opposite was true in the case of employment status (results available upon request).

**Fig. 2:**
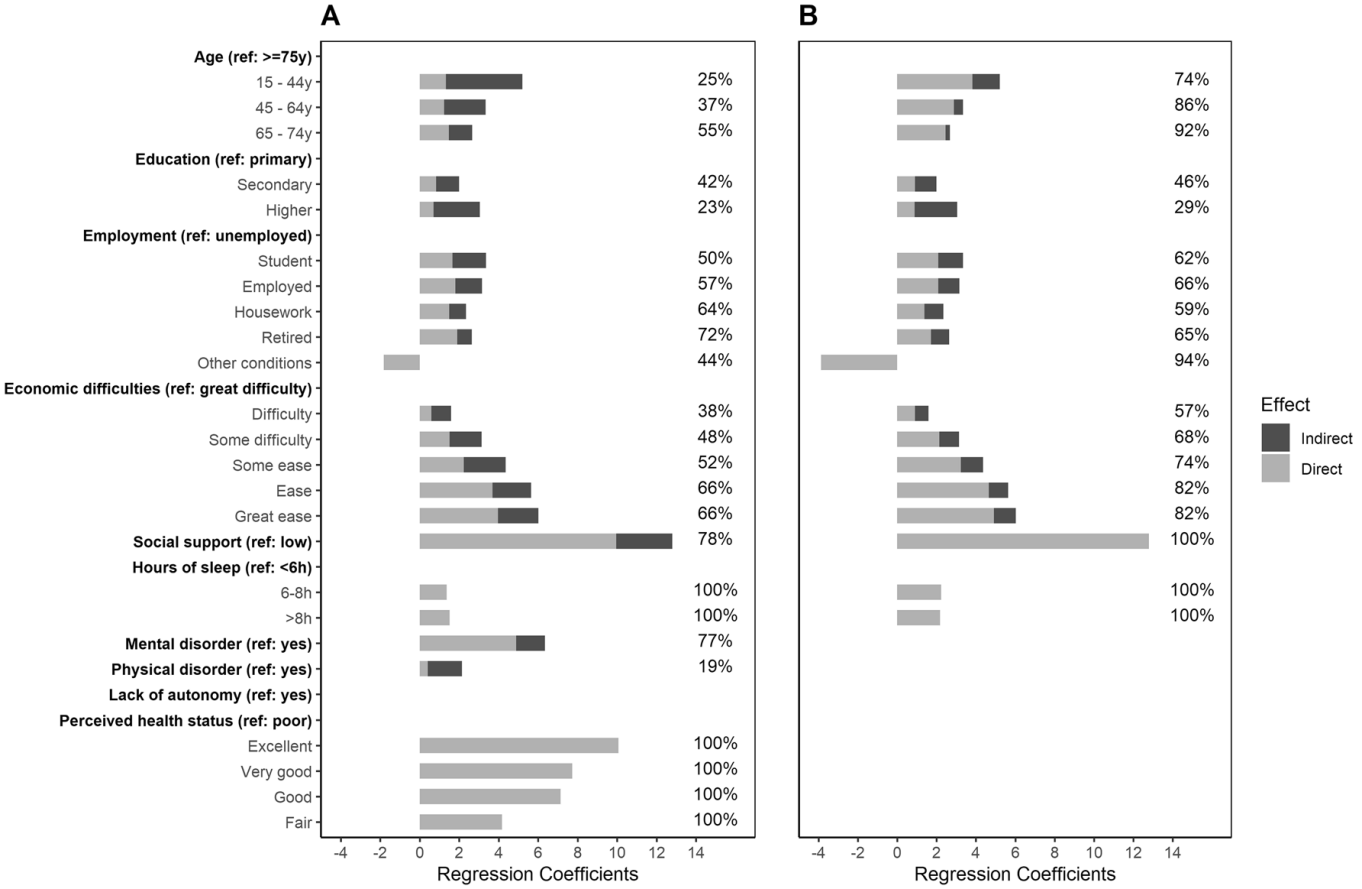
Variable total effects, as regression coefficient value, decomposed into direct (light area) and indirect (dark area). Values adjusted by each variable’s minimally sufficient adjustment set (MSAS) **a** from DAG-1 that includes health factors; and **b** from DAG-2 that excludes health factors. Values over bars represent the percentage of direct effects over total effects. Only variables significantly associated with mental well-being are represented (*P* < 0.05). (Color figure online)

Figure 2a shows total and decomposed (direct and indirect) effects for variable categories on mental well-being. Interestingly, lower levels of well-being due to age were more explained by indirect rather than direct associations: middle-aged subjects (45–64y) presented lower direct scores (*β* = 1.24, SE 0.33) than the younger group (*β* = 1.33, SE 0.36). Contrarily, for socioeconomic factors, direct effects represented about 50% of the total. In fact, the gradient in well-being according to economic difficulties was mostly direct. Regarding relational factors, 77.9% of the total effects came from direct effects. Mental disorders and lack of autonomy showed similar results.

Figure 2b shows total effects (decomposed into direct and indirect effects) when health factors were removed from the DAG to study the contribution of health factors and self-perceived health on the indirect effects of each variable on mental well-being (see Supplementary Table 1 for comparison of direct and total effects when excluding health factors in the DAG-2 and Supplementary Figure 4 for their respective MSAS). The relative contribution of health factors varied across predictors. For example, health factors explained over 65% of the indirect effects of age on well-being and less than 10% of the indirect effect of education. Interestingly, health factors had lower indirect effects on well-being at higher levels of economic difficulties (values ranging from 30 to 50%). Finally, health factors explained all the indirect effects of social support on well-being.

## Discussion

In this study we analysed the association of individual factors and mental well-being. Three main findings emerged: first, differences in the distribution of mental well-being according to gender and age were mostly due to indirect effects, which suggest social inequalities, given that other factors mediate their relationship; second, health factors and self-perceived health were highly associated to mental well-being; third, the lack of perceived functional social support is itself a critical risk factor for lower mental well-being. A novelty of this study is the decomposition of effects for each hypothesis of exposure-outcome. This approach may add valuable information to the study of health disparities and social determinants of health [44].

We found that men had a marginally higher mental well-being than women, in line with previous studies based on WEMWBS [22, 45, 46]. Most of sex association was indirect, via socioeconomic and health factors. This is in contrast with the consistent findings on sex differences in mental health and psychopathology [47, 48]. Our results may imply that sex impacts differently in positive and negative mental states. A similar result was found for age: increasing age was negatively associated with mental well-being, but the effect was mostly indirect and mediated by health factors. Also, age and well-being associations were linear, in contrast with previous studies reporting a U-shape relation, in which young and the elderly people present higher well-being than middle-aged adults [49, 50]. Additional analyses are needed to test potential quadratic effects of age on mental well-being in the population.

Our results revealed associations between economic conditions and mental well-being. Unemployment was a substantial risk factor for lower mental well-being, and a distinct negative gradient in mental well-being appeared according to family economic difficulties. Stewart-Brown *et al*. [22], found that the group with higher economic resource presented better odds of higher mental well-being; however, no differences existed among other groups. In our study, economic effects were direct and had no sizeable mediating role. However, additional analyses showed that, as economic difficulties increased, health factors contributed less to the indirect effect on well-being (see Supplementary Table 1). This result is most relevant when considering that a direct effect of socioeconomic factors on mental well-being in groups with economic difficulties would imply a source of social inequality. Such result aligns with previous research: job type and job context characteristics may determine the relationship between work, income and well-being [51, 52]. Literature also points at less frequent health-oriented behaviours in persons with limited resources, which may contribute to social differences in social well-being [53].

In our study, low functional social support played a key role, and had the most substantial effect in mental well-being scores among all groups. Our findings add to the existing evidence that social support plays a decisive role in the maintenance of psychological well-being and that poor social relationship negatively impact mental health [14, 54, 55]. Moreover, our results suggest that the most important effects of social support on well-being are direct, and their small indirect effect go through health factors. Literature abounds in social support interventions on different outcomes and specific groups [56, 57]. Our findings agree with previous studies showing a strong relationship between physical, psychological health and subjective well-being [17]. Under the model, health factors were the most important contributors to mental well-being. There was also a strong association between self-reported health and mental well-being, those reporting better health also showed higher levels of mental well-being. Qualitative studies show that health is at the core of what constitutes well-being [20]. Suffering from a mental disorder had a huge direct association with well-being, which has been found in previous studies on the relationship between WEMWBS and mental illness [58]. The debate on wether well-being or mental illness are part of the same dual continuum or separate constructs is still open [59]. Our model cannot distinguish whether mental well-being and mental illness are part of the same construct or its outcome. Yet, under our causal assumptions, well-being comes after health factors, so promoting population health may be a way of promoting well-being.

As hypothesized, when decomposing variable effects on well-being through health variables, we found they mediated the associations. This result matches well-known models of disability where it comes as a consequence of health [19, 60, 61]. In spite of potential reverse causality effects between mental well-being and health, our results add to the evidence supporting the sensitivity of mental well-being as a health outcome. In our study, WEMWBS was sensitive to a variety of socioeconomic, relational and health factors. Mental well-being overcomes the disadvantages of instruments designed to be sensitive in the population fraction with mental health problems. This approach potentially capture changes in well-being that otherwise would have gone unnoticed [21, 62]. Such relationships must be explored in future research, ideally in longitudinal studies.

Our results must be interpreted in light of some limitations. Firstly, the temporal ambiguity entailed by cross-sectional designs regarding causality involves that results are as correct as DAG assumptions. It is worth noting that other models may be devised, and that we are not proposing a theoretical framework of mental well-being. Our DAG just intends to systematize [63] and make transparent our assumptions on exposures-outcome relationships. As of date, no theoretical framework exists on mental well-being to guide DAG-building, so we based our model on the widespread and commonly accepted WHO model of determinants of health and disability [19, 60, 61]. Traditional methods based on fit criteria (e.g. *R*^2^ or Akaike information criterion) would take the model as a whole, thus ignoring directionality in the relationships. Secondly, conceptually relevant variables were not available (e.g. social participation [23] or dispositional traits such personality or character variables) [64]). Personality is itself a most relevant variable in behaviour analyses and would indeed affect well-being outcomes. However, mental and physical health are also affected by such variables, so that they might encompass these effects up to a certain point. Finally, the ESCA survey assesses lifetime presence of disorders, so their reporting can either be affected by recall bias or absent at the time of the interview, yielding results which are likely to average both of these influences.

However, this study is not without strengths: its representativeness, sample size, and various variables make it comprehensive and able to detect small effects. Also, the use of MSAS disconnect irrelevant pathways from the multivariable analysis, reducing noise parameter estimates [65]. We have also tested separate models of direct and indirect effects for each exposure-outcome hypothesis, so that results inform of pathways that can be intervened directly on the variable or averted at some point along causal chains. Future models using this kind of effect decomposition may help to refine the study of modifiable effects of health disparities and social determinants of health.

## Conclusions

This study identified a variety of factors associated with the levels of mental well-being, ranging from structural factors such as gender, age and employment, to more proximal factors such as social support and health factors. These results are consistent with previous studies focussing on disability and disease. Our results support that mental well-being, and especially WEMWBS, could be an essential tool for monitoring population health and general well-being. The focus on positive mental health offers an opportunity to expand research on aspects of promotion rather than prevention. Finally, policies aimed at reducing social inequalities are also required to promote the well-being of the population.

## Supplementary Information

Below is the link to the electronic supplementary material.

Supplementary file 1 (DOCX 430 kb)

## Data Availability

The raw data of this study are available from Department of Health of Generalitat de Catalunya. Restrictions apply to the availability of these data, which were used under license for this study. Data that support the findings of this study are available from the corresponding author [GV] with the permission of Department of Health of Generalitat de Catalunya.

## Code availability

The R code is available upon request from the corresponding author.
