# Supplementary material for: Mental well-being of the general population: direct and indirect effects of socioeconomic, relational and health factors
Source: Qual Life Res. 2021 Apr 13;30(8):2171–85. doi: 10.1007/s11136-021-02813-5 (PMC8298347; doi:10.1007/s11136-021-02813-5)
Supplement: Supplementary file 1 — (DOCX 430 kb) [file 11136_2021_2813_MOESM1_ESM.docx]

# Electronic Supplementary Material

**JOURNAL**: Quality of Life Research

**TITLE**: Direct and indirect effects of socioeconomic, relational and health factors on the mental well-being of the general population

**AUTHORS**: Natalia Soldevila-Domenech^1,2,3^†, Carlos G. Forero^4^†, Itxaso Alayo^1,5^, Jordina Capella^6^, Joan Colom^6^, Davide Malmusi^7^, Anna Mompart^8^, Philippe Mortier^1,5^, Beatriz Puértolas^1,5^, Néstor Sánchez^4^, Anna Schiaffino^8,9^, Gemma Vilagut^1,5*,^ Jordi Alonso^1,3,5*^.

^1^Health Services Research Group, Epidemiology and Public Health Program, Hospital del Mar Medical Research Institute (IMIM), Carrer del Doctor Aiguader, 88, PRBB building, 08003 Barcelona, Spain.

^2^Integrative Pharmacology and Systems Neurosciences Research Group, Neurosciences Research Program, Hospital del Mar Medical Research Institute (IMIM), Carrer del Doctor Aiguader, 88, PRBB building, 08003 Barcelona, Spain.

^3^Department of Experimental and Health Sciences, Pompeu Fabra University (UPF), Carrer del Doctor Aiguader, 88, PRBB building, 08003 Barcelona, Spain.

^4^ School of Medicine, Universitat Internacional de Catalunya (UIC), Barcelona, Spain.

^5^CIBER en Epidemiología y Salud Pública (CIBERESP), Av. Monforte de Lemos, 3-5, 28029 Madrid, Spain.

^6^Sub-direcció General de Drogodependències, Agència de Salut Pública de Catalunya, Carrer Roc Boronat, 81-95, 08005 Barcelona, Spain

^7^Ajuntament de Barcelona, Barcelona, Spain.

^8^Direcció General de Planificació en Salut, Departament de Salut, Generalitat de Catalunya, Travessera de les Corts, 131-159, 08028 Barcelona, Spain.

^9^Institut Català d’Oncologia, Gran Via de l’Hospitalet 199-203, 08908 l’Hospitalet de Llobregat, Spain

†Contributed equally. Natalia Soldevila-Domenech and Carlos G. Forero should be consider joint first authors.

*Corresponding authors: Gemma Vilagut and Jordi Alonso should be consider joint senior author.

**Gemma Vilagut**. Health Services Research Group, Epidemiology and Public Health Program, Hospital del Mar Medical Research Institute (IMIM), Carrer del Doctor Aiguader, 88, PRBB building, 08003 Barcelona, Spain. Phone: +34 933 160 745. Email: [gvilagut@imim.es](mailto:gvilagut@imim.es). ORCID-iD: <https://orcid.org/0000-0002-3714-226X>

**Jordi Alonso**. Health Services Research Group, Epidemiology and Public Health Program, Hospital del Mar Medical Research Institute (IMIM), Carrer del Doctor Aiguader, 88, PRBB building, 08003 Barcelona, Spain. Phone: +34 933 160 754. Email: [jalonso@imim.es](mailto:jalonso@imim.es) ORCID iD: <https://orcid.org/0000-0001-8627-9636>


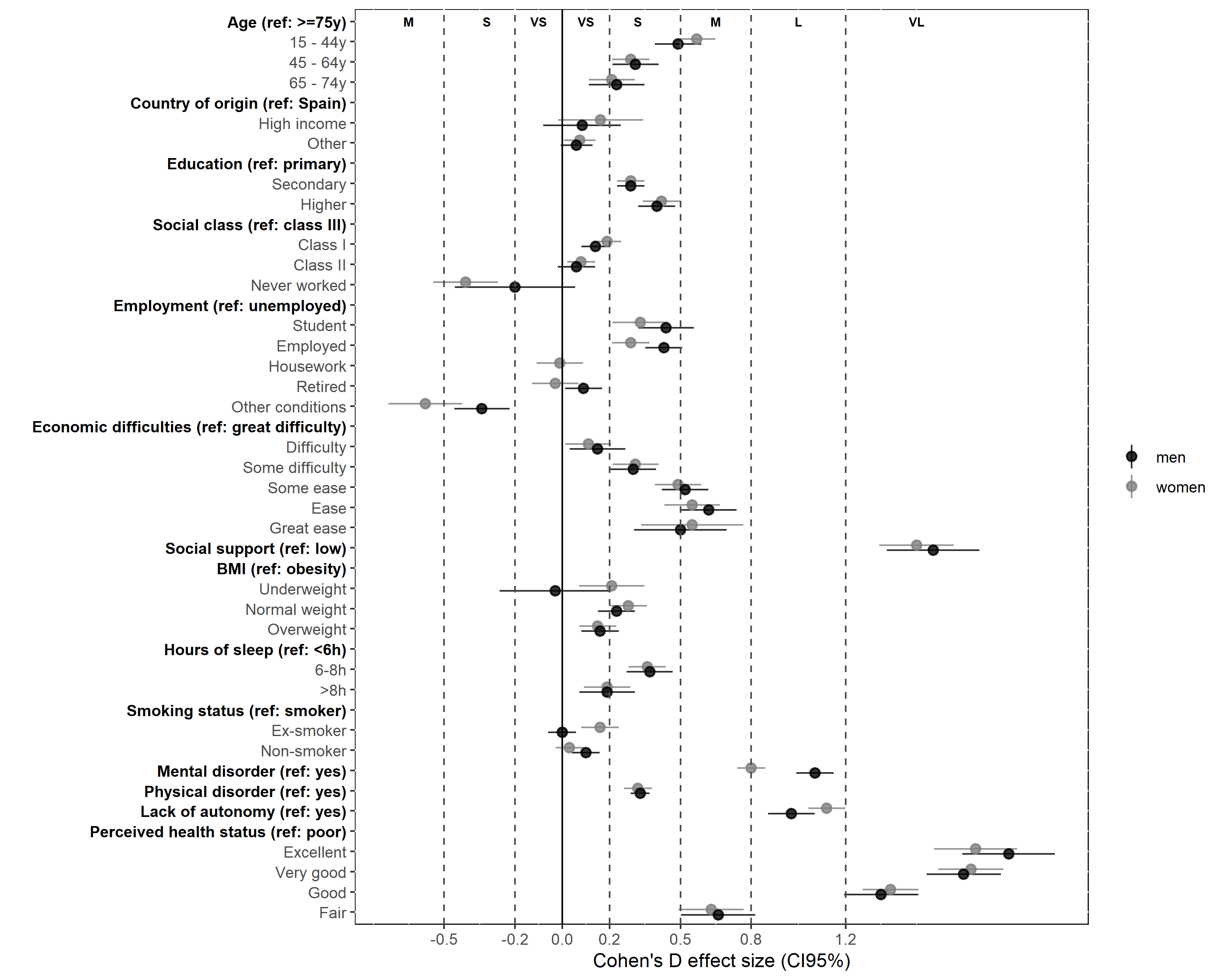


Supplementary Figure 1. Cohen’s D effect of the unadjusted relationship between studied variables and mental well-being


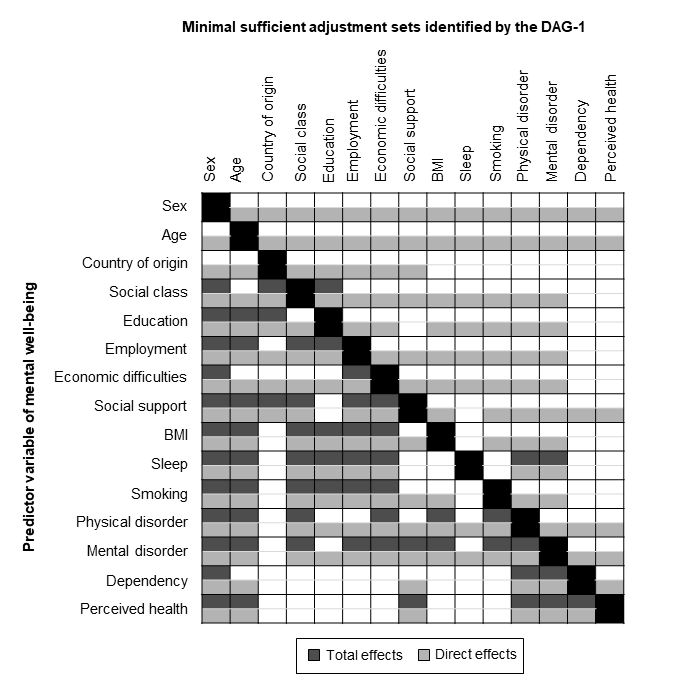


Supplementary Figure 2. Minimally sufficient adjustment set (MSAS) of each predictor variable of mental well-being, identified with the directed acyclic graph (DAG-1, Figure 1) to analyse both total and direct effects.


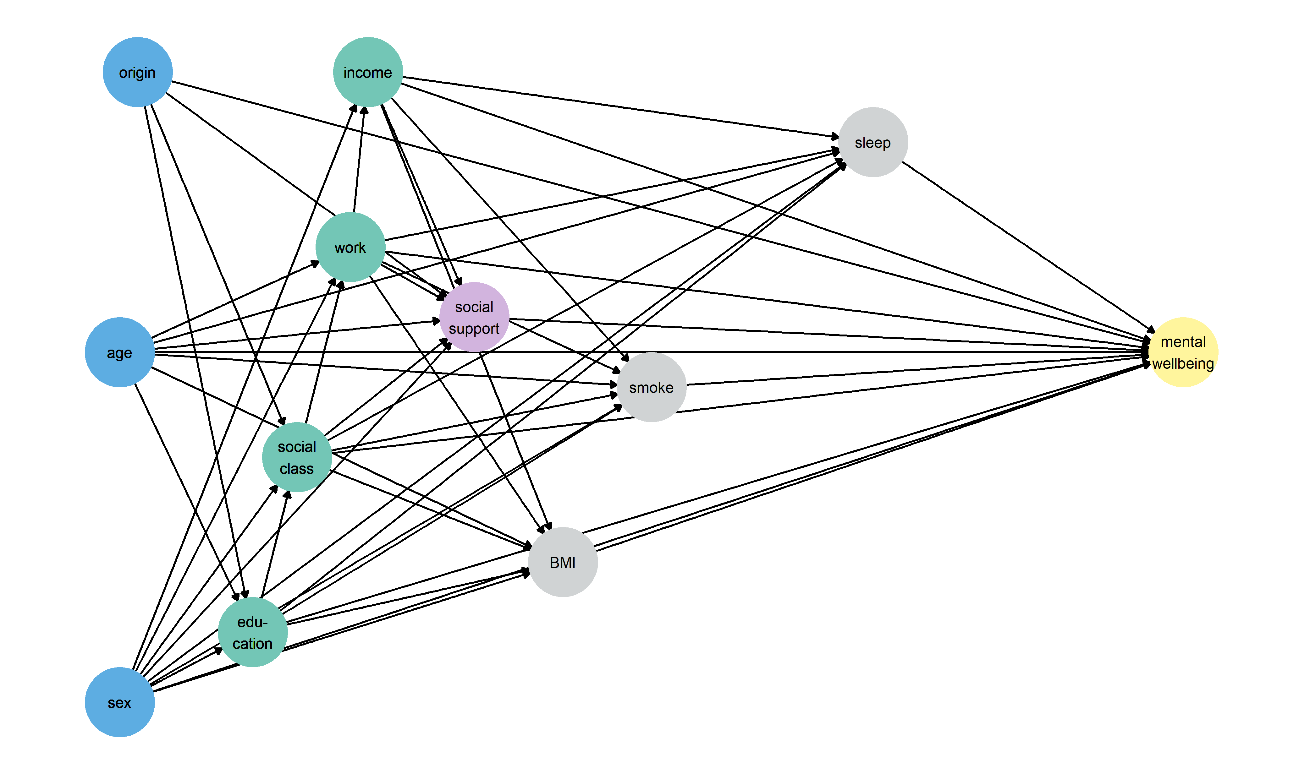


Supplementary Figure 3. Secondary directed acyclic graph (DAG-2) without health factors and perceived health. Variable names are abbreviated: *origin*, country of origin; *education*, educational level; *work*, employment status; *income*, family economic difficulties; *BMI*, body mass index; *smoke*, smoking status; *sleep*, hours of sleep; *dependency*, lack of autonomy. Node colours represent the group to which each variable belongs: demographic factors (in blue), socioeconomic factors (in green) and relational factors (in purple).


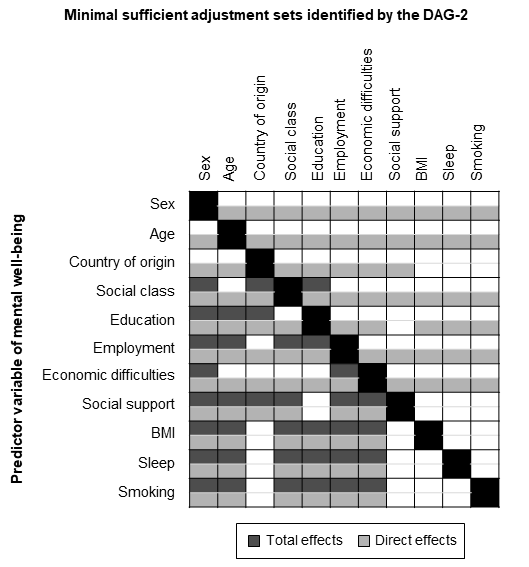


Supplementary Figure 4. Minimally sufficient adjustment set (MSAS) of each predictor variable of mental well-being, identified with the directed acyclic graph (DAG-2, Supplementary Figure 3) to analyse both total and direct effects excluding health factors and perceived health.

**Supplementary Table 1. Comparison of direct and total of each variable on WEMWBS score adjusted by the minimal sufficient adjustment sets identified with DAG-1 that includes health factors and DAG-2 that excludes health factors.**

| **Variable** | **Category** | **DAG-1  (including health factors)** | | | | | | | **DAG-2 (excluding health factors)** | | | | | | **Difference in direct effects** | **Indirect effects explained by health factors*** |
| --- | --- | --- | --- | --- | --- | --- | --- | --- | --- | --- | --- | --- | --- | --- | --- | --- |
|  |  | **Total effects** | | | **Direct effects** | | | **Indirect effects** | **Total effects** | | | **Direct effects** | | |  |  |
|  |  | **β** | **SE** | **P** | **β** | **SE** | **P** |  | **β** | **SE** | **P** | **β** | **SE** | **P** |  |  |
| Sex  (Ref: Women) | Men | 1.32 | 0.15 | <0.001 | -0.05 | 0.14 | 0.741 | NS | 1.32 | 0.15 | <0.001 | 0.95 | 0.15 | <0.001 | NA | NA |
| Age (years)  (Ref: ≥75) | 15 - 44 | 5.21 | 0.26 | <0.001 | 1.33 | 0.36 | <0.001 | 3.89 | 5.21 | 0.26 | <0.001 | 3.83 | 0.38 | <0.001 | 2.51 | 64.51% |
|  | 45 - 64 | 3.35 | 0.27 | <0.001 | 1.24 | 0.33 | <0.001 | 2.10 | 3.35 | 0.27 | <0.001 | 2.89 | 0.36 | <0.001 | 1.64 | 78.14% |
|  | 65 - 74 | 2.67 | 0.32 | <0.001 | 1.47 | 0.29 | <0.001 | 1.19 | 2.67 | 0.32 | <0.001 | 2.46 | 0.31 | <0.001 | 0.99 | 82.82% |
| Country of origin (Ref: Spain) | High income | 1.36 | 0.54 | 0.012 | 0.68 | 0.50 | 0.173 | 0.68 | 1.36 | 0.54 | 0.012 | 0.68 | 0.50 | 0.173 | NA | NA |
|  | Other | 0.90 | 0.21 | <0.001 | 1.01 | 0.21 | <0.001 | -0.11 | 0.90 | 0.21 | <0.001 | 1.01 | 0.21 | <0.001 | 0.00 | 0.00% |
| Educational level (Ref: Primary) | Secondary | 1.99 | 0.20 | <0.001 | 0.84 | 0.19 | <0.001 | 1.15 | 1.99 | 0.20 | <0.001 | 0.91 | 0.20 | <0.001 | 0.07 | 5.87% |
|  | Higher | 3.05 | 0.24 | <0.001 | 0.71 | 0.26 | 0.006 | 2.33 | 3.05 | 0.24 | <0.001 | 0.89 | 0.27 | 0.001 | 0.18 | 7.59% |
| Social class  (Ref: III) | I | 0.68 | 0.22 | 0.002 | -0.42 | 0.20 | 0.037 | 1.10 | 0.68 | 0.22 | 0.002 | -0.45 | 0.21 | 0.032 | -0.03 | -2.68% |
|  | II | 0.16 | 0.21 | 0.444 | -0.46 | 0.18 | 0.013 | 0.62 | 0.16 | 0.21 | 0.444 | -0.45 | 0.19 | 0.021 | 0.01 | 1.90% |
|  | Never worked | -3.75 | 0.52 | <0.001 | -2.58 | 0.50 | <0.001 | -1.17 | -3.75 | 0.52 | <0.001 | -2.30 | 0.53 | <0.001 | 0.28 | -23.76% |
| Employment status  (Ref: Unemployed) | Student | 3.35 | 0.34 | <0.001 | 1.67 | 0.32 | <0.001 | 1.69 | 3.35 | 0.34 | <0.001 | 2.08 | 0.34 | <0.001 | 0.42 | 24.79% |
|  | Employed | 3.16 | 0.24 | <0.001 | 1.81 | 0.23 | <0.001 | 1.35 | 3.16 | 0.24 | <0.001 | 2.08 | 0.24 | <0.001 | 0.27 | 20.07% |
|  | Housework | 2.34 | 0.36 | <0.001 | 1.50 | 0.33 | <0.001 | 0.85 | 2.34 | 0.36 | <0.001 | 1.39 | 0.35 | <0.001 | -0.11 | -12.72% |
|  | Retired | 2.65 | 0.39 | <0.001 | 1.90 | 0.37 | <0.001 | 0.74 | 2.65 | 0.39 | <0.001 | 1.72 | 0.38 | <0.001 | -0.18 | -24.93% |
|  | Other | -4.11 | 0.41 | <0.001 | -1.81 | 0.38 | <0.001 | -2.30 | -4.11 | 0.41 | <0.001 | -3.88 | 0.40 | <0.001 | -2.07 | 90.10% |
| Family economic difficulties to make monthly ends meet  (Ref: Great difficulty) | Difficulty | 1.59 | 0.33 | <0.001 | 0.60 | 0.31 | 0.050 | 0.99 | 1.59 | 0.33 | <0.001 | 0.90 | 0.32 | 0.005 | 0.30 | 30.47% |
|  | Some difficulty | 3.14 | 0.30 | <0.001 | 1.52 | 0.29 | <0.001 | 1.63 | 3.14 | 0.30 | <0.001 | 2.15 | 0.30 | <0.001 | 0.64 | 39.17% |
|  | Some ease | 4.36 | 0.30 | <0.001 | 2.24 | 0.29 | <0.001 | 2.11 | 4.36 | 0.30 | <0.001 | 3.24 | 0.30 | <0.001 | 0.99 | 47.06% |
|  | Ease | 5.64 | 0.33 | <0.001 | 3.70 | 0.32 | <0.001 | 1.95 | 5.64 | 0.33 | <0.001 | 4.65 | 0.34 | <0.001 | 0.95 | 48.93% |
|  | Great ease | 6.02 | 0.55 | <0.001 | 3.98 | 0.52 | <0.001 | 2.05 | 6.02 | 0.55 | <0.001 | 4.91 | 0.55 | <0.001 | 0.93 | 45.42% |
| Social support  (Ref: Low) | Adequate | 12.80 | 0.48 | <0.001 | 9.97 | 0.45 | <0.001 | 2.84 | 12.80 | 0.48 | <0.001 | 12.80 | 0.48 | <0.001 | 2.84 | 100.00% |
| BMI  (Ref: Obesity) | Underweight | -0.38 | 0.50 | 0.446 | -0.80 | 0.46 | 0.086 | NS | -0.38 | 0.50 | 0.446 | -0.38 | 0.50 | 0.446 | NA | NA |
|  | Normal weight | 0.78 | 0.22 | <0.001 | 0.34 | 0.20 | 0.097 | NS | 0.78 | 0.22 | <0.001 | 0.78 | 0.22 | <0.001 | NA | NA |
|  | Overweight | 0.72 | 0.22 | <0.001 | 0.37 | 0.20 | 0.068 | NS | 0.72 | 0.22 | <0.001 | 0.72 | 0.22 | <0.001 | NA | NA |
| Hours of sleep  (Ref: <6h) | 6-8h | 1.37 | 0.24 | <0.001 | 1.37 | 0.24 | <0.001 | 0.00 | 2.24 | 0.26 | <0.001 | 2.24 | 0.26 | <0.001 | NA | NA |
|  | >8h | 1.51 | 0.32 | <0.001 | 1.51 | 0.32 | <0.001 | 0.00 | 2.18 | 0.34 | <0.001 | 2.18 | 0.34 | <0.001 | NA | NA |
| Smoking status  (Ref: smoker) | Ex-smoker | 0.96 | 0.22 | <0.001 | 0.70 | 0.20 | <0.001 | 0.27 | 0.96 | 0.22 | <0.001 | 0.96 | 0.22 | <0.001 | 0.27 | 100.00% |
|  | Non-smoker | 0.65 | 0.18 | <0.001 | 0.31 | 0.16 | 0.058 | NS | 0.65 | 0.18 | <0.001 | 0.65 | 0.18 | <0.001 | NA | NA |
| *It is calculated with the difference in direct effects divided by indirect effects from DAG-1(%).  Each variable has been adjusted by its minimal sufficient adjustment sets identified with the DAG-1 (see Figure 1 and Supplementary Figure 2) or DAG-2 (see supplementary figures 3 and 4), as well as, by the year of survey.  Ref, reference category; β, regression coefficient; SE, standard error; BMI, body mass index. P, p-value. NS, non-significant effect. NA, no apply due to the absence of indirect effects or due to the absence of statistically significant direct effects. | | | | | | | | | | | | | | | | |
